# Atopic Dermatitis in Children: Differential Diagnosis and Mimickers

**DOI:** 10.3390/children13050690

**Published:** 2026-05-18

**Authors:** Beyza Türe Avcı, Tubanur Çetinarslan, Aylin Türel Ermertcan, Regina Fölster-Holst

**Affiliations:** 1 Department of Dermatology, Bursa Inegol State Hospital, 16400 Bursa, Turkey; beyza.tureavci@saglik.gov.tr; 2 Department of Dermatology, Faculty of Medicine, Manisa Celal Bayar University, 45030 Manisa, Turkey; 3 Department of Pediatric Dermatology, Faculty of Medicine, Kiel University, 24105 Kiel, Germany

**Keywords:** pediatric atopic dermatitis, differential diagnosis, age-dependent characteristics

## Abstract

**Highlights:**

**What are the main findings?**

- Atopic dermatitis displays pronounced age-dependent clinical heterogeneity, with distinct morphological and distributional patterns across pediatric age groups.
- Its clinical overlap with a wide range of inflammatory, infectious, and genetic disorders, combined with the lack of specific biomarkers, hindering differential diagnosis.

**What are the implications of the main findings?**

- Recognizing age-specific phenotypes is crucial for accurate diagnosis and reducing diagnostic delay or misclassification.
- A systematic, pattern-based diagnostic approach and the exclusion of age-specific conditions that may clinically mimic the disease are essential for optimizing clinical decision-making and improving patient outcomes.

**Abstract:**

**Background:** Atopic dermatitis (AD) is a chronic, relapsing inflammatory dermatosis that is characterized by pruritus, xerosis, and age-dependent clinical heterogeneity. Accurately diagnosing AD remains challenging due to the absence of specific biomarkers and the broad spectrum of conditions that may mimic its presentation. A wide range of inflammatory, infectious, and genetic disorders resemble AD, including seborrheic dermatitis, psoriasis, contact dermatitis, scabies, dermatophytosis, and nummular eczema, as well as rare immunodeficiency and metabolic conditions. This review summarizes the evolution of the clinical features of pediatric AD across infancy, childhood, and adolescence, with a focus on key differential diagnoses. Recognizing age-specific patterns and potential mimickers is essential for improving diagnostic accuracy and guiding appropriate management in pediatric AD. **Methods**: This study was designed as a narrative review. A structured literature search was conducted of PubMed/MEDLINE for studies published between January 2001 and March 2026 using predefined keywords related to AD, childhood, diagnosis, and differential. Clinical trials, randomized controlled trials, systematic reviews, meta-analyses, and guidelines or consensus documents were included. Studies focusing exclusively on adults or lacking clinical relevance were excluded. A qualitative synthesis was performed due to the heterogeneity in study designs and outcomes. **Results:** This review demonstrates that pediatric atopic dermatitis exhibits marked age-dependent clinical heterogeneity, with distinct morphological features and lesion distribution patterns across infancy, childhood, and adolescence. Furthermore, the substantial clinical overlap with a broad spectrum of inflammatory, infectious, and genetic disorders—combined with the absence of specific diagnostic biomarkers—significantly complicates accurate differential diagnosis and increases the risk of misclassification. **Conclusions**: The recognition of age-specific patterns and potential mimickers is essential for improving diagnostic accuracy and guiding appropriate management in pediatric AD.

## 1. Introduction

Atopic dermatitis [AD] is a chronic, relapsing inflammatory skin disorder characterized by pruritus, xerosis, and eczematous lesions, with heterogeneous clinical manifestations. It is one of the most common dermatologic diseases in childhood and represents a considerable burden for affected children and their families due to its chronic course and impact on quality of life [1,2,3]. Epidemiological studies have estimated that AD affects approximately 10–20% of children worldwide, with most cases beginning at 3–6 months of age [2,4]. Nearly half of patients develop symptoms within the first months after birth, and up to 90% present before the age of five years [2,5].

Studies have shown that although the disease starts early in life, a substantial proportion of patients exhibit a chronic or relapsing course that may persist into adolescence and adulthood, or the disease may present de novo in adulthood. Longitudinal observations indicate that ongoing disease activity beyond childhood is not uncommon and is associated with factors such as early onset and concomitant atopic conditions [6].

AD is a complex inflammatory disorder arising from the interaction of genetic susceptibility, environmental influences, epidermal barrier defects, and immune dysregulation. A hallmark of its pathogenesis is the predominance of type 2 immune responses, largely mediated by interleukin (IL)-4 and IL-13, which activate the JAK–STAT signaling pathway, promote Th2 polarization, and enhance IgE synthesis alongside eosinophilic inflammation. Beyond their immunomodulatory roles, these cytokines critically disrupt skin barrier function by reducing the expression of key structural proteins such as filaggrin, loricrin, and involucrin, thereby increasing transepidermal water loss and facilitating allergen entry. Concurrently, impaired antimicrobial defense mechanisms and shifts in the cutaneous microbiome, particularly increased *Staphylococcus aureus* colonization further sustains inflammation. Moreover, IL-4 and IL-13 contribute to pruritus via neuroimmune interactions and play a role in tissue remodeling, which together drive the chronic, relapsing, and clinically diverse nature of AD [7].

AD is associated with multiple comorbidities, including allergic diseases such as asthma, allergic rhinitis, and food allergy, as well as infectious complications related to impaired skin barrier function [6]. Cutaneous infections are particularly frequent, with *Staphylococcus aureus* colonization reported in up to 90–100% of patients and contributing to disease severity and exacerbations [8]. In addition, patients are predisposed to viral infections such as eczema herpeticum, which may lead to severe systemic involvement due to defective cutaneous immunity [2,8].

The burden of AD extends beyond physical symptoms, significantly impairing the quality of life of both patients and their families, with reported effects including sleep disturbance, activity limitation, and psychosocial stress [8]. Furthermore, AD has been associated with impaired school performance, reduced self-esteem, and broader social and family dysfunction [2].

The diagnosis of AD remains primarily clinical using standardized diagnostic frameworks, particularly the Hanifin and Rajka criteria, which include major features such as pruritus, typical morphology and distribution, chronicity, and personal or family history of atopy, and are supported by additional minor criteria [2,6]. These minor criteria encompass a range of non-specific findings, including xerosis, elevated serum IgE levels, early age of onset, tendency toward cutaneous infections, stigmata as Dennie–Morgan infraorbital folds, palmar hyperlinearity, and pityriasis alba [2].

Laboratory findings can provide supportive information in selected cases. Elevated total IgE levels and peripheral eosinophilia are frequently observed in patients with AD and may correlate with disease severity; however, these findings are non-specific and are neither sensitive nor diagnostic on their own [1,2,6]. Furthermore, allergen-specific IgE testing may indicate sensitization to environmental or food allergens, but positive results do not necessarily indicate clinically relevant allergy and should therefore be interpreted cautiously within the clinical context [2]. In most cases, an early food allergy is of transient nature.

Despite these characteristic features and laboratory findings, the diagnosis of AD can be challenging because there are no pathognomonic findings or specific biomarkers. Over the past few years, emerging noninvasive diagnostic tools such as ‘dermoscopy’ have gained importance in enhancing diagnostic accuracy and differentiating AD from its mimickers. Nevertheless, dermoscopy alone is insufficient for establishing a definitive diagnosis. Although several ancillary methods—such as serum IgE measurement, blood eosinophil levels and skin biopsy—can aid in diagnosis, clinical evaluation remains the cornerstone of diagnosis, and numerous conditions must be considered in the differential diagnosis. Given the need for studies that systematically classify the disorders included in the differential diagnosis of AD according to age group, the present study examined the relevant conditions and recommended diagnostic investigations across three categories: infancy, childhood, and adolescence [9].

This article provides an overview of the clinical course of AD across age groups, highlighting age-related variations in lesion distribution and morphology; emphasizing the clinical scenarios in which additional diagnostic investigations—such as skin biopsy, potassium hydroxide (KOH) examination, dermoscopy, patch testing, and genetic analyses—may be warranted to support differential diagnosis; and providing a structured diagnostic algorithm to guide clinicians in distinguishing AD from its mimickers.

## 2. Materials and Methods

This study was designed as a narrative review aiming to summarize the differential diagnosis and age-related characteristics of AD. This review primarily focused on clinically relevant studies addressing age-related symptoms, diagnosis and differentiating AD from its potential mimickers. The study selection process was performed using a PRISMA-like flow diagram to enhance transparency, while reflecting the narrative nature of the review rather than a formal systematic review design.

**Figure 1:**
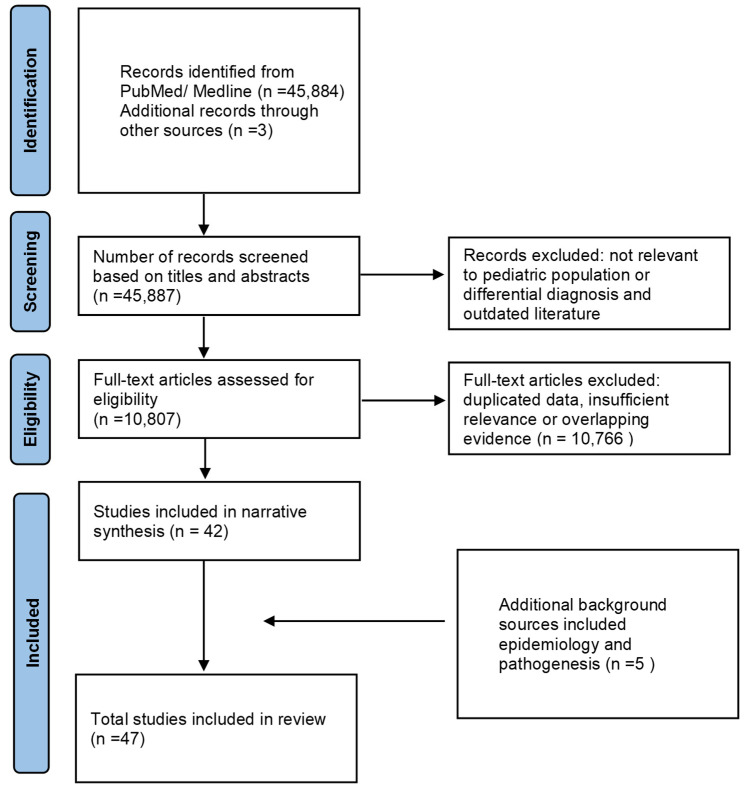
Flow Diagram of the Literature Search and Selection Process.

A structured literature search was performed of the PubMed/MEDLINE database. The search included studies published from January 2001 to March 2026. The following search terms were used in different combinations: [“atopic dermatitis” OR “atopic eczema”] AND [“infant” OR “childhood” OR “adolescence” OR “pediatric”] AND [“diagnosis” OR “differential” OR “diagnostic approach”]. The filters applied during the search included English language, human studies, and child age group [birth–18 years]. Article types were limited to clinical trials, systematic reviews, meta-analyses, narrative reviews, case reports, and guideline or consensus documents. In addition to database searching, relevant international guidelines and consensus statements related to AD diagnosis and differentials were identified through a manual search of reference lists and guideline repositories. Studies were excluded if they focused exclusively on adult populations, unrelated dermatologic conditions, treatment modalities, or evaluating responses to treatments as were outdated/relatively old studies (Figure 1).

## 3. Infantile Atopic Dermatitis

Infantile AD represents the earliest clinical stage of the disease and demonstrates characteristic morphological and distributional features that differ from those observed in later childhood and adolescence [6,10]. The development of early disease is closely associated with epidermal barrier dysfunction and immune dysregulation, which contribute to increased transepidermal water loss, cutaneous dryness, and enhanced susceptibility to environmental triggers [6,11]. As a result of these underlying mechanisms, xerosis and increased skin reactivity are often among the earliest dermatologic findings observed in infants with AD [4,6].

**Figure 2:**
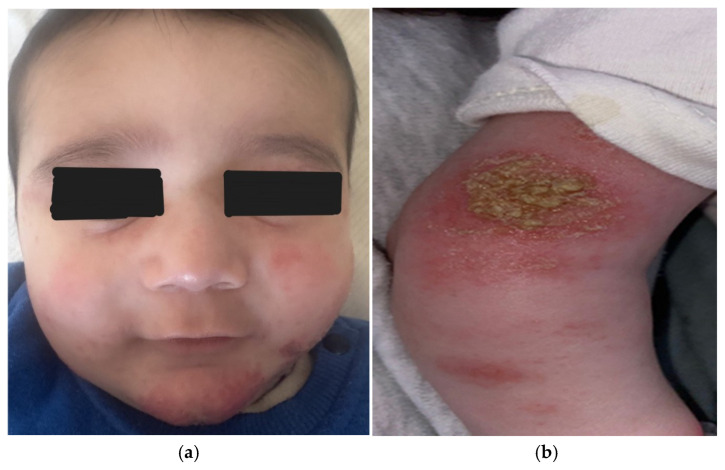
(**a**) Dennie–Morgan infraorbital folds in a child with eczematous plaques on the face. (**b**) An impetiginized plaque on the extensor surface of the arm of a patient with AD.

Clinically, lesions mostly appear on the cheeks [Figure 2a] and scalp and may extend to the trunk and extensor surfaces of the extremities [Figure 2b] during disease flares [5,10]. Acute eruptions typically present as erythematous papules and plaques, accompanied by vesiculation, serous exudation, and crust formation, reflecting the predominantly inflammatory nature of the early disease [5,10]. Pruritus is a hallmark feature in infancy, although infants often express itching indirectly through irritability, sleep disturbance, and repeated rubbing of affected skin against clothing or bedding [2]. Continuous mechanical irritation may lead to excoriations and promote secondary bacterial colonization, particularly by *Staphylococcus aureus*, which frequently colonizes the skin of patients with AD and may exacerbate cutaneous inflammation [2,10].

The distribution of skin lesions provides an important diagnostic clue in infants with suspected AD [5,12]. Facial involvement combined with the relative sparing of the diaper area is frequently observed and may help distinguish AD from other dermatoses commonly encountered during infancy [5]. Nevertheless, those with infantile AD may exhibit considerable clinical heterogeneity, and several morphological variants, including follicular, papular, and nummular patterns, have been described [3,5]. In rare situations, early AD may present as generalized erythema or erythroderma, which require careful evaluation to exclude other dermatologic or systemic disorders, including life-threatening diseases, as primary immunodeficiency syndromes [3].

The differential diagnosis of infantile AD is broad and includes inflammatory, infectious, and systemic conditions, which may show similar clinical findings [Table 1] [3,12]. Seborrheic dermatitis represents one of the most frequent mimickers because it often occurs during the same age period and also involves the scalp and facial regions [5,12,13]. However, seborrheic dermatitis is usually characterized by oilier scales and minimal pruritus, whereas AD typically presents with marked pruritus and xerosis [12]. In addition, seborrheic dermatitis commonly affects intertriginous areas and the diaper region, which are typically spared in infantile AD [5].

**Figure 3:**
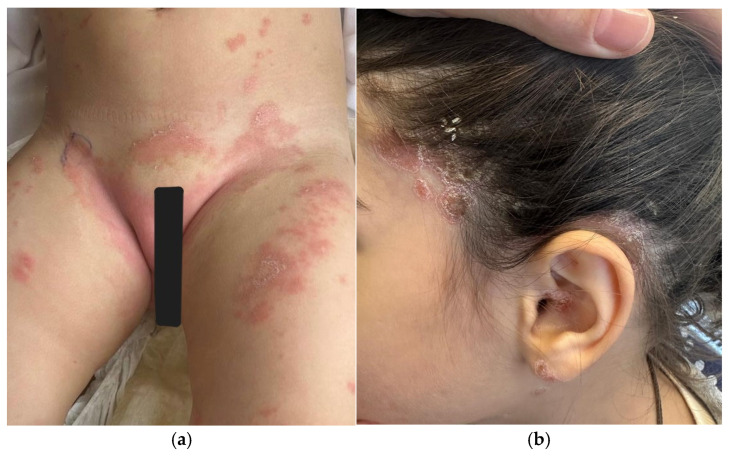
(**a**) The involvement of the diaper area in an infant diagnosed with psoriasis. (**b**) Scaly erythematous plaques on scalp in a child with psoriasis.

Psoriasis is another inflammatory condition that should be included in the differential diagnosis of AD [12]. Infantile psoriasis presents with sharply demarcated erythematous plaques and frequently involves the diaper region [Figure 3a,b], unlike AD. In addition, dermoscopy serves as a valuable noninvasive tool in the differential diagnosis between psoriasis and AD by revealing distinct vascular and morphological patterns. Dermoscopy of psoriasis is typically characterized by uniformly distributed glomerular or dotted vessels, whereas AD demonstrates a dull red–pinkish background with diffusely distributed dotted vessels, often accompanied by yellow serocrusts and/or white scales [14].

Contact dermatitis usually presents as localized reactions associated with irritants—most notably diaper dermatitis—or allergens [12,15,16]. In infants, diaper dermatitis is typically limited to the diaper area and results from exposure to irritants such as moisture, urine, and feces, whereas AD usually spares the diaper region and presents as pruritic eczematous lesions on other body sites [17].

**Figure 4:**
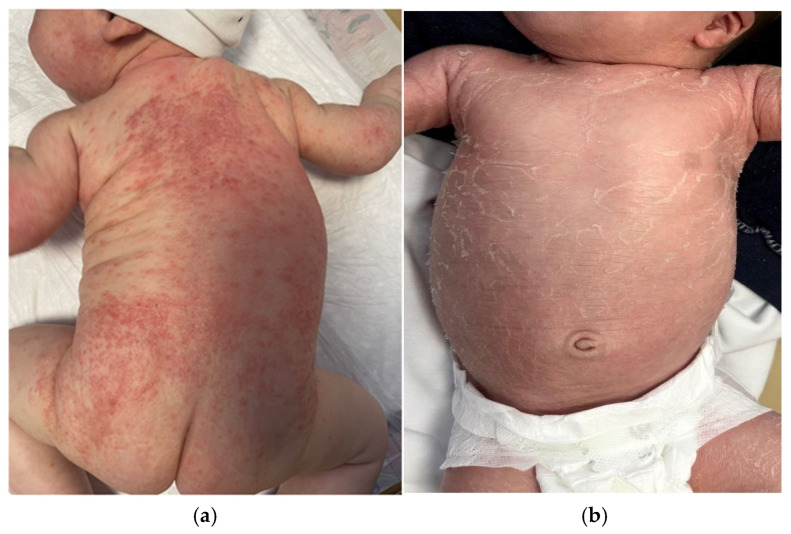
(**a**) Scabies mimicking atopic dermatitis in a newborn. (**b**) Ichthyoses, characterized by diffuse scaling and erythema in a newborn.

Infectious causes such as scabies infestation may also mimic AD and should be suspected when pruritus is severe or when lesions involve the palms, soles, or axillary folds [13,18] [Figure 4a]. Additionally, a history of pruritus affecting other household members and the nocturnal exacerbation of itching may raise suspicion for scabies, while the dermoscopic identification of burrows and a mite can serve as valuable diagnostic clues in the differential diagnosis [19]. In cases with clinical suspicion of scabies, the isolation of the parasite and its identification by microscopic examination are also diagnostic [20].

Dermatophyte infections and candidiasis may cause eczema-like eruptions; however, characteristic findings such as annular plaques or satellite pustules may aid in distinguishing these disorders [12,17]. In equivocal cases, potassium hydroxide (KOH) examination —the wet-mount preparations of skin scrapings from the active border of the lesion demonstrating hyphal elements or yeast forms—is also helpful in the differential diagnosis [21].

In some cases, severe or atypical eczematous lesions during infancy may indicate an underlying genetic or systemic disease rather than classic AD [3]. Conditions such as acrodermatitis enteropathica, metabolic diseases like phenylketonuria, and certain primary immunodeficiency disorders [Netherton syndrome, Wiskott–Aldrich syndrome, Omenn syndrome, and hyper-IgE syndrome] often present as eczematous lesions resembling AD during early life [3,12].

Acrodermatitis enteropathica is a rare inherited zinc absorption disorder caused by mutations affecting intestinal zinc transport, typically presenting in infancy with periorificial and acral dermatitis, diarrhea, and alopecia [22]. In contrast to AD, which usually manifests as pruritic eczematous lesions with a flexural distribution, acrodermatitis enteropathica characteristically involves periorificial areas [around the mouth, eyes, and anus] and acral sites, accompanied by systemic findings such as diarrhea and hair loss, which are not typical findings of AD [12,22].

Phenylketonuria may mimic AD due to the presence of eczematous skin changes; however, it can be distinguished by the associated neurologic impairment, hypopigmentation, and metabolic abnormalities related to phenylalanine accumulation [12]. Measurements of plasma phenylalanine levels and genetic testing are valuable for confirming the diagnosis [1].

Internal organ involvement with hepatosplenomegaly and lymphadenopathy as well as generalized eczematous lesions without the localizing features of infantile AD suggest immunodeficiency syndromes [12,23,24]. Netherton syndrome should be suspected in infants with eczematous dermatitis resembling AD when the disease is accompanied by neonatal or early-onset erythroderma, impaired growth, recurrent infections, and hair shaft abnormalities such as trichorrhexis invaginata, with confirmation achieved via identifying pathogenic mutations in the SPINK5 gene [25,26]. Wiskott–Aldrich syndrome may resemble AD due to the presence of early-onset eczematous skin lesions; however, it can be distinguished by the associated immunodeficiency features, particularly recurrent infections, thrombocytopenia with small platelets, and bleeding tendency [3]. Omenn syndrome may also mimic severe AD due to the diffuse eczematous dermatitis and elevated IgE levels; however, it is distinguished by early-onset erythroderma, lymphadenopathy, hepatosplenomegaly, and severe immunodeficiency [27]. Similarly, DOCK8 deficiency may mimic severe AD because of the early-onset eczema and markedly elevated IgE levels; however, it can be differentiated from AD by the recurrent severe viral infections, increased susceptibility to malignancies, and underlying combined immunodeficiency [27].

In the infantile period, distinguishing ichthyosis from AD may be challenging because both conditions can present as generalized xerosis and scaling. However, ichthyosis typically manifests as persistent, diffuse scaling [Figure 4b] from early life, without the characteristic eczematous inflammation and intense pruritus commonly seen in AD. In contrast, AD in infants more often presents as erythematous, pruritic eczematous lesions, and a relapsing course [5,12].

## 4. Childhood Atopic Dermatitis

During childhood, the clinical phenotype of AD evolves and differs from the typical pattern observed during infancy [6,10]. In children between two and twelve years of age, lesions most frequently involve flexural areas, particularly the antecubital fossae, popliteal fossae, wrists, ankles, and neck [5,10]. The chronic inflammation and persistent scratching during this stage often result in lichenified plaques and excoriations, reflecting the long-standing nature of the disease [2,10].

In addition to flexural lesions, children with AD frequently exhibit generalized xerosis and may develop characteristic associated findings such as Dennie–Morgan infraorbital folds, pityriasis alba, keratosis pilaris, and palmar hyperlinearity [6,10,11]. Recurrent disease flares and chronic pruritus may significantly impair sleep quality and daily functioning in affected children [28]. The distribution of lesions in childhood AD is strongly influenced by the chronic itch–scratch cycle and mechanical irritation, which contribute to the predominance of eczema in flexural areas such as the antecubital and popliteal fossae [2,10].

**Figure 5:**
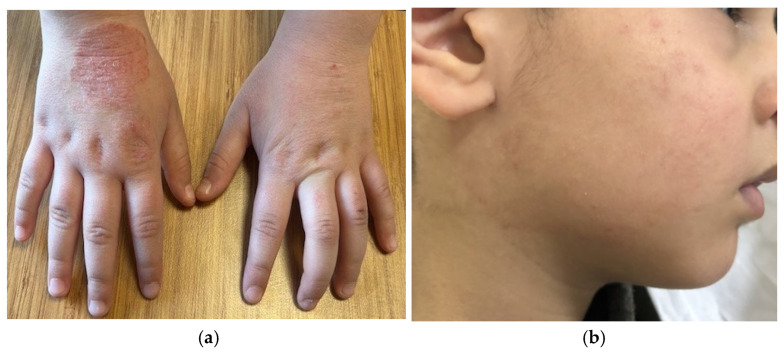
(**a**) Nummular eczema. Pruritic coin-shaped erythematous plaques on hand. (**b**) Tinea faciei. Erythematous papules with central clearing on cheeks.

Despite the relatively characteristic distribution pattern, the clinical morphology of childhood AD is variable, and several dermatologic disorders should be considered in the differential diagnosis [12]. Contact dermatitis is important in the differential diagnosis, particularly when lesions appear in areas exposed to potential irritants or allergens and demonstrate a well-demarcated distribution corresponding to contact with external substances [12]. For example, exposure to nickel-containing metal snaps on the waistband of jeans can result in localized eczematous or lichenoid dermatitis beneath the umbilicus, representing a classic presentation of allergic contact dermatitis (ACD) to nickel in school-aged children [29]. We must also consider contact allergy in patients with AD who do not respond to treatment according to guidelines. In these cases, the patch test is helpful. Nummular eczema is an inflammatory dermatosis characterized by multiple coin-shaped eczematous plaques, most often on the legs. It is a clinically described term and a manifestation of diverse diseases such as AD and contact dermatitis, and it is also linked to infection, dry skin, and alcohol intake [30,31]. Nummular eczema may be pruritic, scaly, and dry [Figure 5a]. In pediatric cases, the condition rarely persists beyond puberty [3,12].

Infectious conditions may further complicate the diagnostic process. Tinea corporis may produce annular erythematous lesions with peripheral scaling that occasionally resemble the eczematous plaques in AD [3,12]. Tinea capitis [Figure 5b] may appear as subtle, uninflamed plaques, potentially mimicking infantile seborrheic dermatitis or AD. Diagnostic differentiation can be supported by the identification of alopecia, pustules, lymphadenopathy, and broken hair shafts as well as dermoscopy [3,9,32]. As previously noted, the diagnosis can be clarified by potassium hydroxide (KOH) examination [21]. Scabies infestation should also be considered when pruritus is intense, particularly if the lesions involve the interdigital spaces, wrists, or axillary regions or when other family members report similar symptoms [3,32]. The diagnosis of scabies can be supported by dermoscopic and microscopic examinations [20].

Psoriasis presents with sharply demarcated erythematous plaques and silvery scales, which may help distinguish it from the more poorly defined eczematous lesions of AD. Additionally, the involvement of extensor areas, the retroauricular area, the anal fold, and the umbilical area helps to differentiate psoriasis from childhood AD [3]. In this age group, although the predominance of AD lesions in flexural areas may aid in the clinical differential diagnosis, dermoscopic evaluation and, in cases with a persistent suspicion of psoriasis, skin biopsy can be employed to establish a definitive diagnosis [33].

Other inflammatory dermatoses may also mimic childhood AD. Molluscum contagiosum may trigger an eczematous reaction surrounding viral lesions, a phenomenon commonly referred to as molluscum dermatitis [3,12]. Children may scratch the affected areas and inadvertently remove the molluscum lesions. Because the small papules can be difficult to recognize within inflamed skin and pruritus is prominent, these cases are often misdiagnosed as AD. It remains unclear whether molluscum-associated dermatitis occurs predominantly in individuals with underlying AD [12]. Dermoscopy is also a useful tool in the differential diagnosis, in which molluscum contagiosum typically shows central umbilication with white-to-yellow polylobular amorphous structures surrounded by crown vessels that spare the central area [34]. Periorificial dermatitis should be considered when erythematous papulopustules occur around the mouth and eyes, particularly in children who have used topical corticosteroids [12].

**Figure 6:**
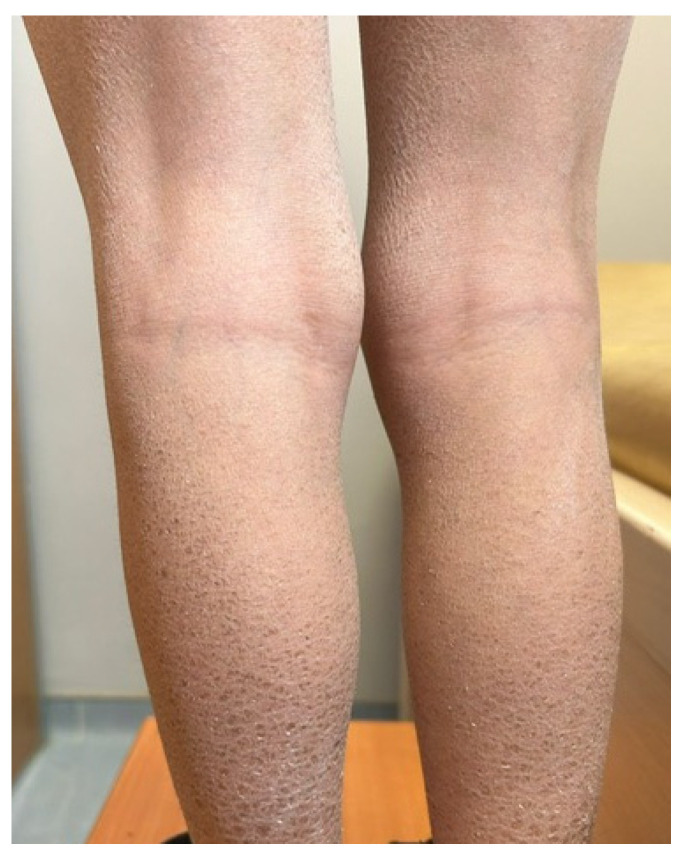
Ichthyosis vulgaris. Diffuse scaling with mild pruritus on both legs.

Several rare genetic syndromes can manifest as cutaneous findings during childhood and may mimic AD; hereditary alpha tryptasemia [HAT] is among these conditions. HAT is a genetic condition caused by increased copy numbers of the TPSAB1 gene, leading to elevated basal serum tryptase levels and multisystem manifestations including flushing, urticaria, pruritus, and other mast-cell-mediated symptoms. Although pruritic and eczematous skin findings may resemble AD, HAT can be differentiated by persistently elevated basal tryptase levels and systemic mast-cell-related symptoms such as flushing, gastrointestinal complaints, and anaphylaxis [35]. In addition, certain genetic syndromes [Figure 6] and metabolic disorders, previously discussed in the context of infancy, may also be included in the differential diagnosis of AD during childhood, as diagnostic delays can lead to their later clinical recognition.

Because several conditions resemble childhood AD, accurate diagnosis requires the careful evaluation of lesion morphology, anatomical distribution, associated symptoms, and patient and family history [3,12].

## 5. Adolescent Atopic Dermatitis

Adolescent AD represents a later stage of the disease in which the clinical phenotype evolves from the patterns typically observed in infancy and childhood [6,10]. During adolescence, lesions often become more localized and chronic, frequently demonstrating pronounced lichenification as a result of persistent inflammation and repeated scratching. The diagnosis of AD in adolescents remains primarily clinical and is based on characteristic lesion morphology, a chronic relapsing disease course, and the typical anatomical distribution of eczema [2,6].

**Figure 7:**
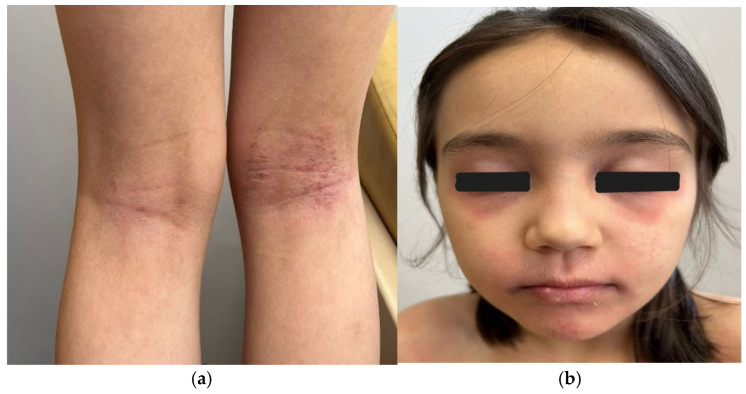
(**a**) Plaques in both popliteal fossae, presenting with chronic lichenification and pruritus. (**b**) Periocular involvement in child with AD.

**Figure 8:**
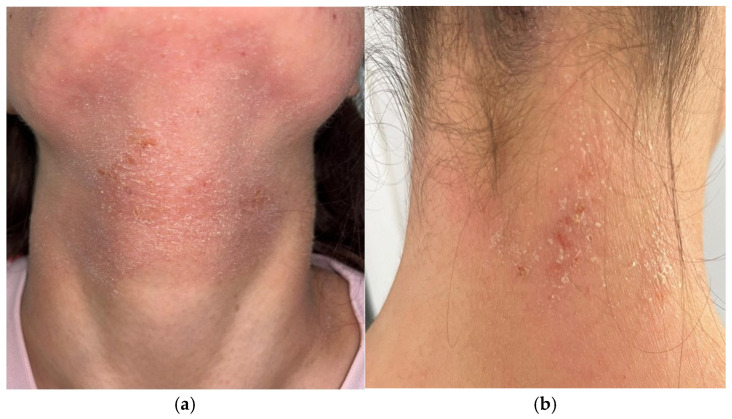
Erythematous scaly plaques on the (**a**) anterior and (**b**) posterior surfaces of the neck in adolescent patients with AD.

The distribution of lesions in adolescents most commonly involves flexural areas such as the antecubital and popliteal fossae [Figure 7] as well as the neck [Figure 8] and periocular regions [6]. In addition to flexural eczema, adolescents may develop dermatitis affecting the head region and the hands, reflecting environmental exposure and repeated mechanical irritation of the skin [3,6,10]. Chronic lesions are frequently characterized by thickened plaques, excoriations, and accentuated skin markings caused by long-standing pruritus and repetitive scratching [2,10].

Generalized xerosis and increased skin sensitivity are also commonly observed in adolescents with AD and contribute to recurrent disease flares [6,11]. In contrast to the more exudative lesions typically observed during infancy, adolescent AD is usually characterized by dry, lichenified plaques reflecting chronic inflammatory skin disease [2,10]. The head-and-neck variant of AD is particularly recognized in this age group and may be associated with environmental allergens, microbial colonization, or irritant exposures [3,6].

A number of dermatologic disorders should be considered in the differential diagnosis of adolescent AD. ACD is particularly relevant during adolescence because the exposure to metals, cosmetics, fragrances, and topical medications becomes increasingly common in this age group [12]. ACD should be considered in the differential diagnosis of AD, as ACD can coexist with AD and act as a factor that worsens disease severity [3,32]. Lesions of ACD are often sharply demarcated, presenting with vesicular lesions in the acute stage and xerotic crusting in the chronic stage, corresponding to areas of allergen exposure, which may help differentiate the disease from the more diffuse pattern typically observed in AD [36]. In treatment-resistant cases, particularly in this age group, patch testing is recommended when ACD is suspected [32,37].

Seborrheic dermatitis may also resemble AD when affecting the scalp, eyebrows, or nasolabial folds. However, seborrheic dermatitis generally presents as greasy scaling and less-intense pruritus compared with AD [1,12]. Dermoscopy may serve as a useful adjunct in this differential diagnosis. The dermoscopic findings of seborrheic dermatitis are characterized by dotted vessels distributed in a patchy pattern along with yellowish scales; in addition, indistinct linear branching vessels and whitish scales may be observed [38]. Like in other pediatric age, groups psoriasis should also be considered in adolescence and usually manifests as well-demarcated erythematous plaques covered with silvery scale, commonly involving the scalp and extensor surfaces [1,3]. Psoriasis is frequently misdiagnosed as AD, as it tends to manifest as more subtle, less-well-demarcated plaques and reduced scaling compared to adult psoriasis. Furthermore, approximately 5% of children exhibit clinical overlap between AD and psoriasis. This differential diagnosis can be challenging due to the often subtle and atypical presentation of psoriasis in pediatric patients. Notably, psoriasis should be considered in cases of dermatitis who demonstrate an unusual resistance to conventional therapeutic interventions [15,32,39].

**Figure 9:**
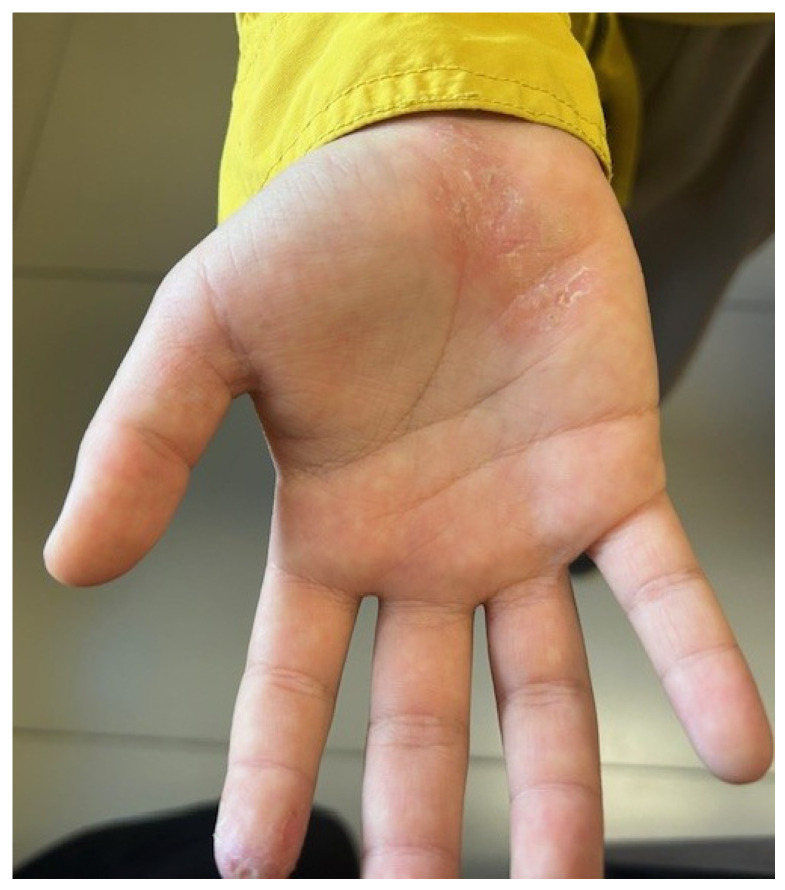
Erythematous, scaly, fissured plaque localized on palmar surface.

Chronic hand eczema [Figure 9] represents another important differential diagnosis, particularly in adolescents with predominant hand involvement and a history of repeated exposure to irritants [3,40]. Chronic hand eczema not only represents an important differential diagnostic consideration for AD but also occurs with increased frequency on an AD background, suggesting a strong pathogenic and clinical association between these conditions [41]. The most significant endogenous risk factors include AD and xerosis. A meta-analysis published in 2018 demonstrated that AD is associated with a three- to fourfold increase in both the one-year and lifetime prevalence of hand eczema and that up to one-third of individuals with active hand eczema report a history of AD [42,43]. Notably, more than half of the patients with active AD develop hand involvement [41]. As such, chronic hand eczema plays a pivotal role in the differential diagnosis. In cases of hand eczema that are refractory to standard therapeutic approaches, particularly when contact allergy is strongly suspected, patch testing should be undertaken as part of the diagnostic workup [44].

In rare cases, persistent or atypical eczematous lesions during adolescence may represent the early manifestations of cutaneous T-cell lymphoma, which should be suspected when lesions are unusual in appearance or resistant to standard treatment [3,8]. Hypopigmented mycosis fungoides [MFs], the most prevalent pediatric form of cutaneous T-cell lymphoma [CTCL], can closely resemble the pityriasis alba commonly observed in patients with AD [45]. Clinically, it manifests as poorly defined, hypopigmented patches with minimal scaling, predominantly localized to photo-protected areas such as the bathing suit distribution. These lesions may be pruritic and display fine wrinkling, attributable to epidermal atrophy [46]. The careful evaluation of lesion morphology, anatomical distribution, clinical history, and response to therapy is therefore essential for distinguishing the two conditions. The definitive diagnosis is established through skin biopsy, which typically indicates a lymphocytic infiltrate with prominent epidermotropism; immunohistochemical evaluation and T-cell receptor gene rearrangement analysis can provide additional diagnostic support [3].

Table 2 summarizes the clinical features and differential diagnosis of AD according to age group.

## 6. Conclusions

AD in childhood represents a diagnostically challenging and clinically heterogeneous entity, characterized by dynamic, age-dependent variations in lesion morphology and distribution. As highlighted throughout this review, the differential diagnosis of pediatric AD is remarkably broad, encompassing a wide spectrum of inflammatory, infectious, genetic, immunologic, and metabolic disorders that may closely mimic AD presentations.

In this context, the reliance on clinical pattern recognition alone may be insufficient for diagnosis, particularly in atypical, treatment-resistant, or early-onset cases. Therefore, a structured and integrative diagnostic approach is essential [Table 3]. While detailed history-taking and careful physical examination remain the cornerstones of diagnosis, adjunctive diagnostic tools play a critical role in refining the differential diagnosis. Laboratory parameters such as the total IgE and eosinophil levels may support the presence of atopic predisposition but lack specificity. In contrast, targeted investigations—including potassium hydroxide [KOH] examination for fungal infections, dermoscopy for psoriasis, scabies or seborrheic dermatitis, and patch testing in suspected allergic contact dermatitis—provide valuable, context-dependent diagnostic clues.

Importantly, in cases with severe, atypical, or syndromic features, advanced diagnostic modalities such as genetic and metabolic testing should be considered to exclude underlying genodermatoses, immunodeficiency syndromes, or metabolic disorders. Similarly, skin biopsy remains a key diagnostic tool in selected cases, particularly when inflammatory dermatoses such as psoriasis or rare conditions including cutaneous T-cell lymphoma are suspected.

Ultimately, improving the diagnostic accuracy of pediatric AD requires not only an awareness of its diverse clinical phenotypes but also a high suspicion of mimickers across different age groups. The judicious use of ancillary diagnostic methods, guided by the clinical context, is crucial to avoid misdiagnosis, ensure appropriate management, and prevent unnecessary or potentially harmful treatments. A comprehensive, age-adapted, and multimodal diagnostic strategy thus forms the foundation for optimal care in children with suspected atopic dermatitis.

## Figures and Tables

**Table 1:** Differential diagnosis of atopic dermatitis in infancy [3,5].

**Disease**	**Distinguishing Clinical Features from AD**	**Diagnostic Tests**
Seborrheic dermatitis	Oily, yellow scales; minimal pruritus; diaper/intertriginous involvement	Clinical; dermoscopy if needed
Psoriasis	Well-demarcated plaques; diaper involvement; scaling	Dermoscopy; biopsy if needed
Contact dermatitis	Localized; diaper area; exposure history	Clinical; patch testing
Scabies	Nocturnal pruritus; palms/soles; family history	Dermoscopy; microscopy
Tinea	Annular plaques; central clearing	KOH examination; culture
Candidiasis	Satellite pustules; diaper area	KOH examination
Acrodermatitis enteropathica	Periorificial dermatitis; diarrhea; alopecia	Serum zinc levels; genetic testing
Phenylketonuria	Eczema + neurologic signs; hypopigmentation	Metabolic tests (phenylalanine levels)
Wiskott–Aldrich syndrome	Eczema + recurrent infections + thrombocytopenia (small platelets) + bleeding tendency	Immunologic work-up; genetic testing
DOCK8 deficiency	Severe eczema + recurrent viral infections + high IgE + malignancy risk	Immunologic evaluation; genetic analysis
Netherton syndrome	Erythroderma + hair shaft defects (*trichorrhexis invaginata*) + failure to thrive	Genetic testing (SPINK5); microscopy of hair
Omenn syndrome	Early erythroderma + lymphadenopathy + hepatosplenomegaly + severe immunodeficiency	Immunologic work-up; genetic testing
Hyper-IgE syndrome	Severe eczema-like dermatitis + recurrent infections + markedly elevated IgE	Serum IgE; genetic testing
Ichthyosis	Diffuse scaling; minimal inflammation; no marked pruritus	Clinical evaluation; genetic testing

**Table 2:** Age-dependent clinical characteristics of atopic dermatitis in pediatric patients [1,3,6].

	Typical Distribution of Lesions	Morphology/Clinical Features	Common Associated Findings	Important Differential Diagnoses
**Infantile AD [0–2 years]**	Cheeks, scalp, forehead, extensor surfaces of arms and legs; trunk may be involved; diaper area usually spared	Erythematous, exudative plaques; vesicles, crusting and oozing lesions; intense pruritus; acute inflammatory appearance	Irritability and sleep disturbance due to itching; xerosis; possible secondary bacterial infection	Seborrheic dermatitis, irritant diaper dermatitis, candidiasis, infantile psoriasis, scabies, immunodeficiency disorders, ichthyosis
**Childhood AD [2–12 years]**	Flexural areas including antecubital fossae, popliteal fossae, wrists, ankles, and neck	Lichenified plaques caused by chronic scratching; excoriations; persistent xerosis; chronic relapsing eczema	Dennie–Morgan folds, pityriasis alba, keratosis pilaris, palmar hyperlinearity	Contact dermatitis, nummular eczema, tinea corporis, scabies, psoriasis, molluscum dermatitis, perioral dermatitis
**Adolescent AD [≥12 years]**	Flexural areas may persist; periorbital areas, head, neck region, hands, and sometimes nipples	Chronic lichenified plaques; hand eczema, head–neck dermatitis; localized persistent lesions	Eyelid eczema, nipple dermatitis, prurigo-like lesions, psychosocial impact	Allergic contact dermatitis, seborrheic dermatitis, psoriasis, chronic hand eczema, rarely cutaneous T-cell lymphoma

**Table 3:** Diagnostic approach to pediatric atopic dermatitis and its mimickers.

Step	Clinical Scenario	Action/Diagnostic Test
Start	Child with pruritic eczematous lesions	→Clinical assessment (history, morphology, distribution, age]
	Typical features of AD?	
YES	Diagnose AD	→Initiate AD-directed therapy
NO/Atypical	Infectious causes suspected Tinea (annular plaques with central clearing] Scabies (nocturnal pruritus, family history, burrows] Candida (satellite pustules]	→KOH/culture →Dermoscopy ± microscopy →KOH
	Psoriasis suspected (well-demarcated, scaly plaques; knees, scalp, elbows]	→Dermoscopy ± biopsy
	Allergic contact dermatitis (suspicious exposure history]	→Patch testing
	Genetic syndromes (Wiskott–Aldrich, DOCK8, Netherton]	→Genetic testing
	Metabolic disease (phenylketonuria, acrodermatitis enteropathica]	→Phenylalanine/zinc levels
	Mycosis fungoides (pruritic hypopigmented patches in sun-protected areas]	→Skin biopsy
	Assess response to treatment	
YES	Adequate response	→Continue AD care
NO	Treatment-resistant	→Reconsider diagnosis → Return to differential pathway → Advanced testing/biopsy

## Data Availability

Data results are available upon reasonable request from the corresponding author.

## Abbreviations

The following abbreviations are used in this manuscript:

AD	Atopic Dermatitis
ACD	Allergic Contact Dermatitis
HAT	Hereditary Alpha Tryptasemia
CTCL	Cutaneous T-Cell Lymphoma
MF	Mycosis Fungoides
